# Correction: ATX-2, The *C*. *elegans* Ortholog of Human Ataxin-2, Regulates Centrosome Size and Microtubule Dynamics

**DOI:** 10.1371/journal.pgen.1006519

**Published:** 2016-12-27

**Authors:** Michael D. Stubenvoll, Jeffrey C. Medley, Miranda Irwin, Mi Hye Song

The reference for the origin of the strain WM210 is missing in S1 Table. The reference is: Gnazzo MM, Uhlemann EM, Villarreal AR, Shirayama M, Dominguez EG, Skop AR. The RNA-binding protein ATX-2 regulates cytokinesis through PAR-5 and ZEN-4. Mol Biol Cell. 2016 Oct 15;27(20):3052–3064. Epub 2016 Aug 24. Interested researchers should use this information to request the WM210 strain. Please view the correct S1 Table below.

## Supporting Information

S1 Table List of *C*. *elegans* strains in this study. (DOCX)

## References

[1] Stubenvoll MD, Medley JC, Irwin M, Song MH (2016) ATX-2, the C. elegans Ortholog of Human Ataxin-2, Regulates Centrosome Size and Microtubule Dynamics. PLoS Genet 12(9): e1006370 doi: 10.1371/journal.pgen.1006370 27689799 10.1371/journal.pgen.1006370 PMC5045193

