# Supplementary figures and images for: ATX-2, the C. elegans Ortholog of Human Ataxin-2, Regulates Centrosome Size and Microtubule Dynamics
Source: PLoS Genet. 2016 Sep 30;12(9):e1006370. doi: 10.1371/journal.pgen.1006370 (PMC5045193; doi:10.1371/journal.pgen.1006370)

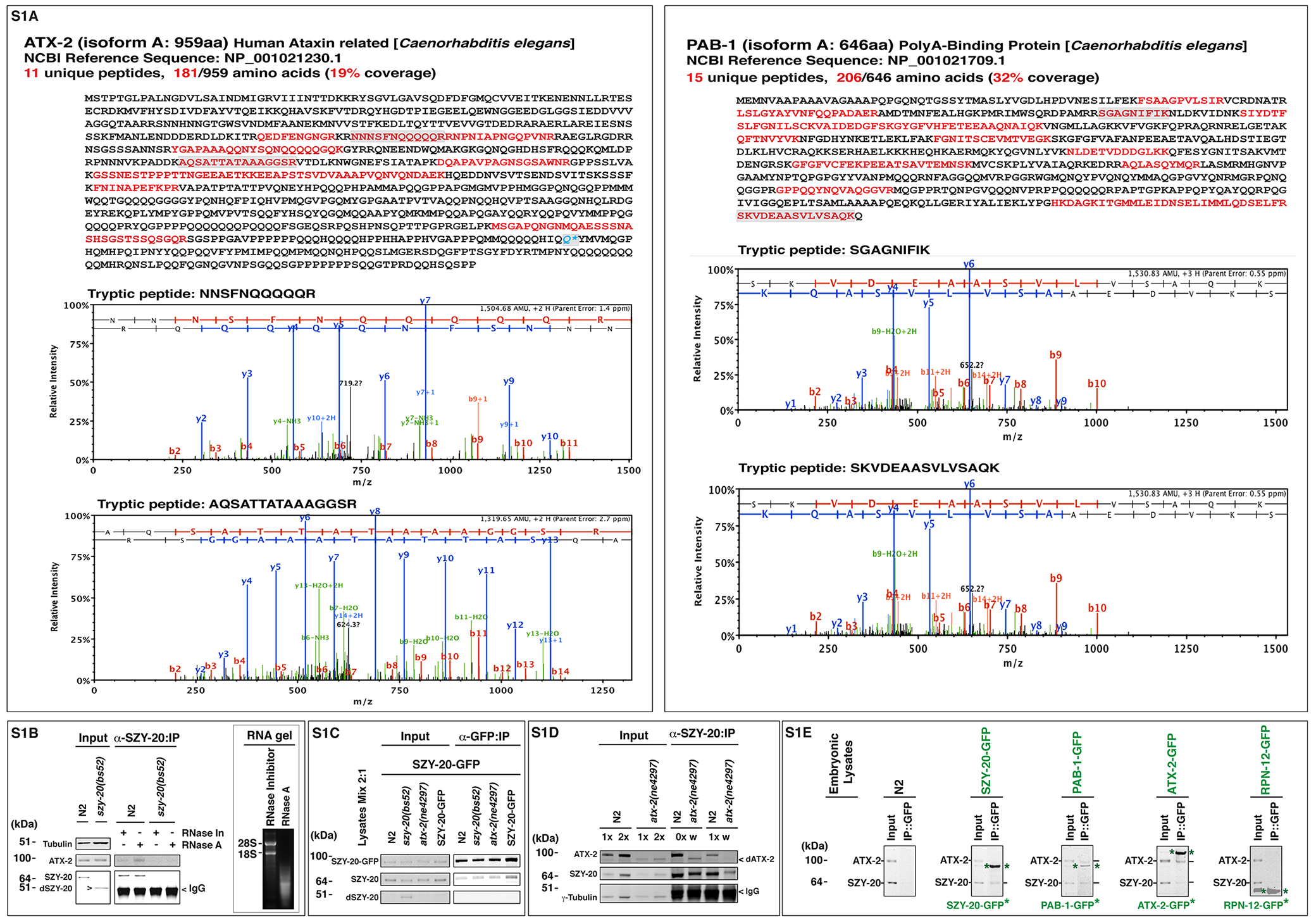

Supplement: S1 Fig — (A) The amino acid sequences of peptides identified by MS/MS analysis and matched to those of ATX-2 and PAB-1 are highlighted in red. In comparison, MS analyses of the control IP showed only 2% peptide coverage for ATX-2 and 2.3% for PAB-1. The representative MS/MS spectra of two selected tryptic peptides (indicated in grey box) for each protein are shown below. In ATX-2, Q* in blue indicates the position that is mutated to stop codon in the atx-2(ne4297) strain (CAG to TAG), resulting in 128 aa deletion at the C-terminus. (B) ATX-2 is undetectable in pull-down from szy-20(bs52) lysates, while ATX-2 co-precipitates with SZY-20 in both RNase inhibitor or RNaseA treated wild-type lysates. Total RNAs were extracted from lysates treated with either RNase Inhibitor or RNase A using TRIzol (Thermo Fisher), analyzed by agarose gel electrophoresis: RNase A treatment efficiently degraded RNAs in lysates, whereas lysates treated with RNase Inhibitor exhibit bands of 18S and 28S ribosomal RNAs. In pull-down, the truncated form (dSZY-20 >) of SZY-20 in szy-20(bs52) mutants is possibly masked due to heavy detection of IgG heavy chain (<) near 51kDa. (C) To avoid heavy IgG detection, we performed α-GFP based IP assay using SZY-20-GFP embryonic lysates mixture with N2, szy-20(bs52), atx-2(ne4297) lysates. The truncated form of SZY-20 (dSZY-20) is undetectable in pull-down from SZY-20-GFP embryonic lysates, while the full-length SZY-20 co-precipitates with SZY-20-GFP, suggesting that the C-terminus of SZY-20 is required for the complex formation. (D) In atx-2(ne4297), the C-terminal truncation of ATX-2 (dATX-2) alleviates its interaction with SZY-20. dATX-2 signal is dramatically diminished in SZY-20 co-precipitates after 1x wash (1x w), compared to no wash (0x w) following IP, suggesting that the C-terminus of ATX-2 influences physical interaction with SZY-20. (E) Pull-down assays with α-GFP, using embryonic lysates from a strain expressing GFP fused to the C-terminus of SZY-20, P [file pgen.1006370.s001.tif]

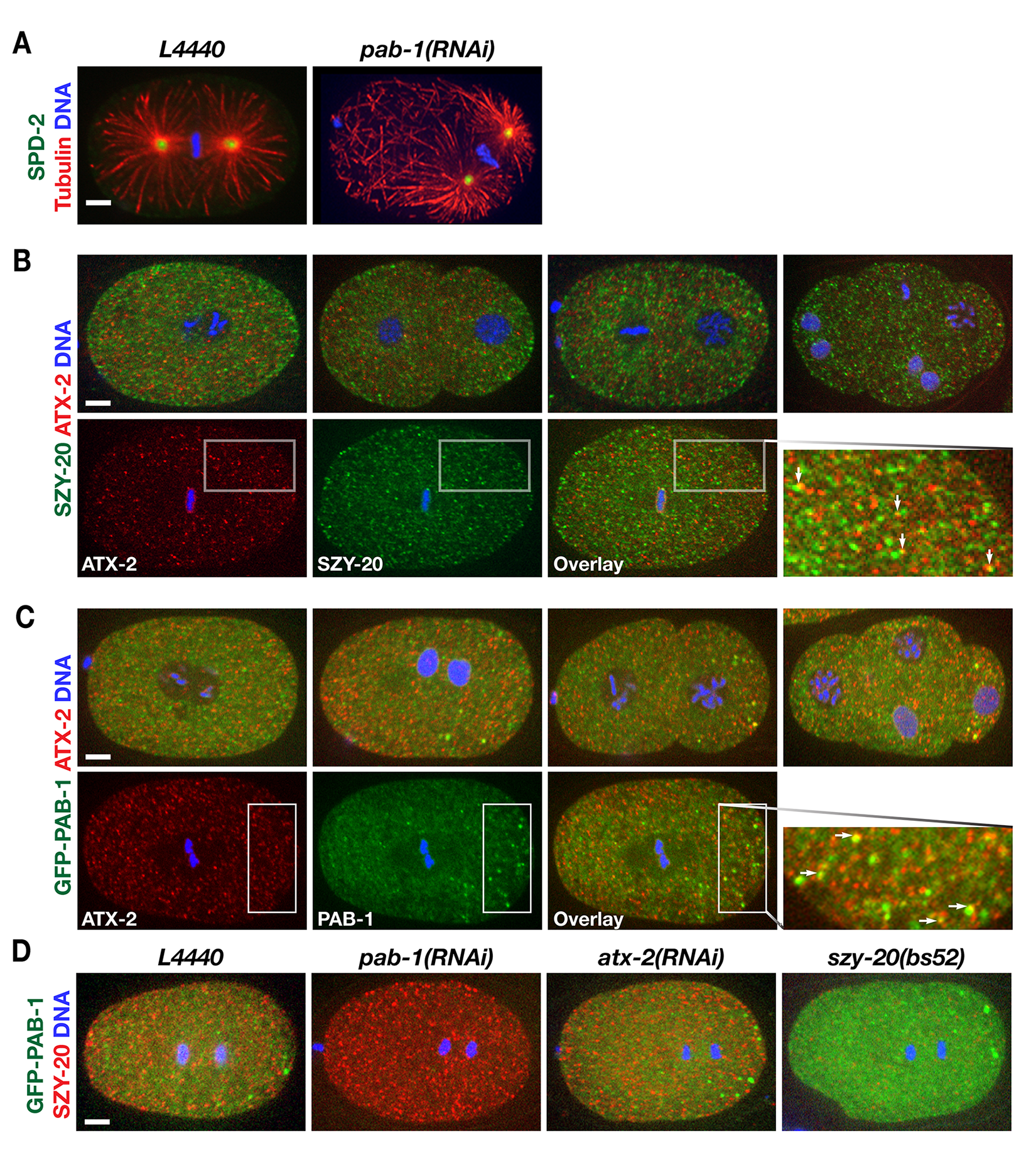

Supplement: S2 Fig — (A) pab-1(RNAi) results in abnormal spindle positioning. (B) Wild-type embryos co-labeled for ATX-2 and SZY-20 illustrate that only a small fraction (~13%, n = 11) of ATX-2 and SZY-20 foci coincide in the cytoplasm in early embryos (top). The embryo in Fig 3C is shown in each channel and the overlay (bottom). Arrows indicate colocalization. (C) Embryos co-labeled for ATX-2 and GFP-PAB-1 illustrate that cytoplasmic foci of ATX-2 and PAB-1 partially coincide (~20%, n = 13) (top). The embryo in Fig 3D is shown in each channel and the overlay (bottom). Arrows indicate co-localizing cytoplasmic foci. (D) GFP-PAB-1 expressing embryos co-stained for GFP and SZY-20 illustrate that GFP-PAB-1 and SZY-20 partially colocalize (~3%, n = 3) in the cytoplasm, and that SZY-20 expression is unaffected by atx-2 or pab-1(RNAi). Colocalization was quantified using the MetaMorph software. Circular regions with a diameter of 60 pixels were drawn at different positions within the cytoplasm. Threshold intensity values were set for each wavelength, and the percentage of overlapping pixels from each wavelength above the defined threshold values was calculated. Boxes for the overlays are magnified (2–2.5x) views. Bars, 5 μm. (TIF) [file pgen.1006370.s002.tif]

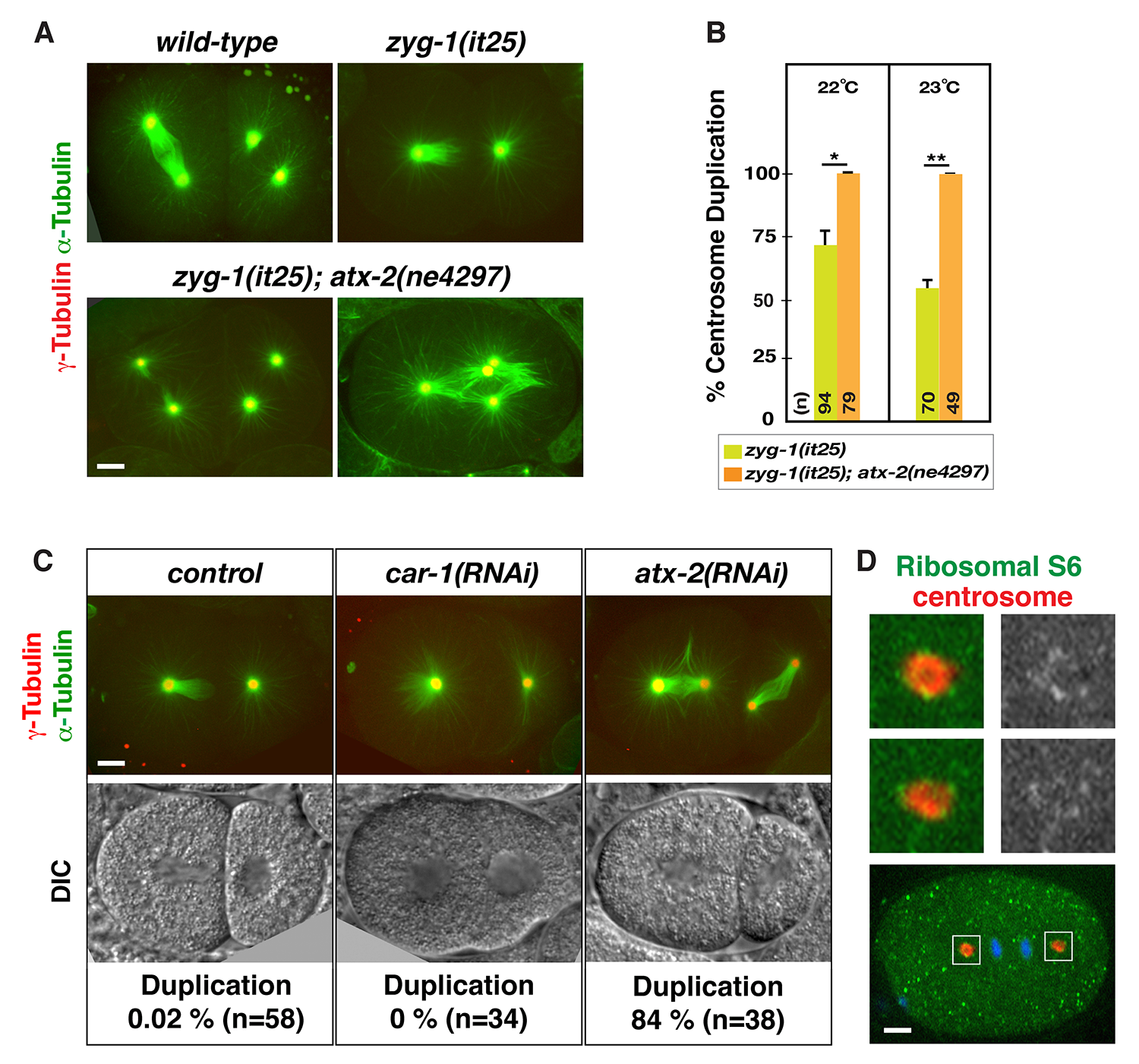

Supplement: S3 Fig — (A-B) atx-2(ne4297) mutation restores bipolar spindle formation to zyg-1(it25) embryos. At the second cell cycle, zyg-1(it25); atx-2(ne4297) double homozygous mutant embryos exhibits a cytokinesis failure with tetra-poles, indicating successful centrosome duplication. (B) Quantification of centrosome duplication based on the bipolar spindle formation at 22 and 23°C, semi-restrictive conditions for zyg-1(it25). (C) Depleting CAR-1, an RNA-binding protein, does not restore centrosome duplication to zyg-1(it25) mutants. It is shown that CAR-1, another known RNA-binding protein, is required for normal cytokinesis [36, 37]. At 24°C, car-1(RNAi) in zyg-1(it25) results in 100% of monopolar spindles at the second mitosis, while atx-2(RNAi) restores > 80% of centrosome duplication to zyg-1(it25). Thus, RNA-binding proteins ATX-2 and SZY-20 play a specific role in negatively regulating centrosome duplication. (D) Ribosomal subunit S6 partially coincides with γ-Tubulin at centrosomes in wild-type embryos. Insets are magnified 5-fold. Bar, 5 μm. (TIF) [file pgen.1006370.s003.tif]

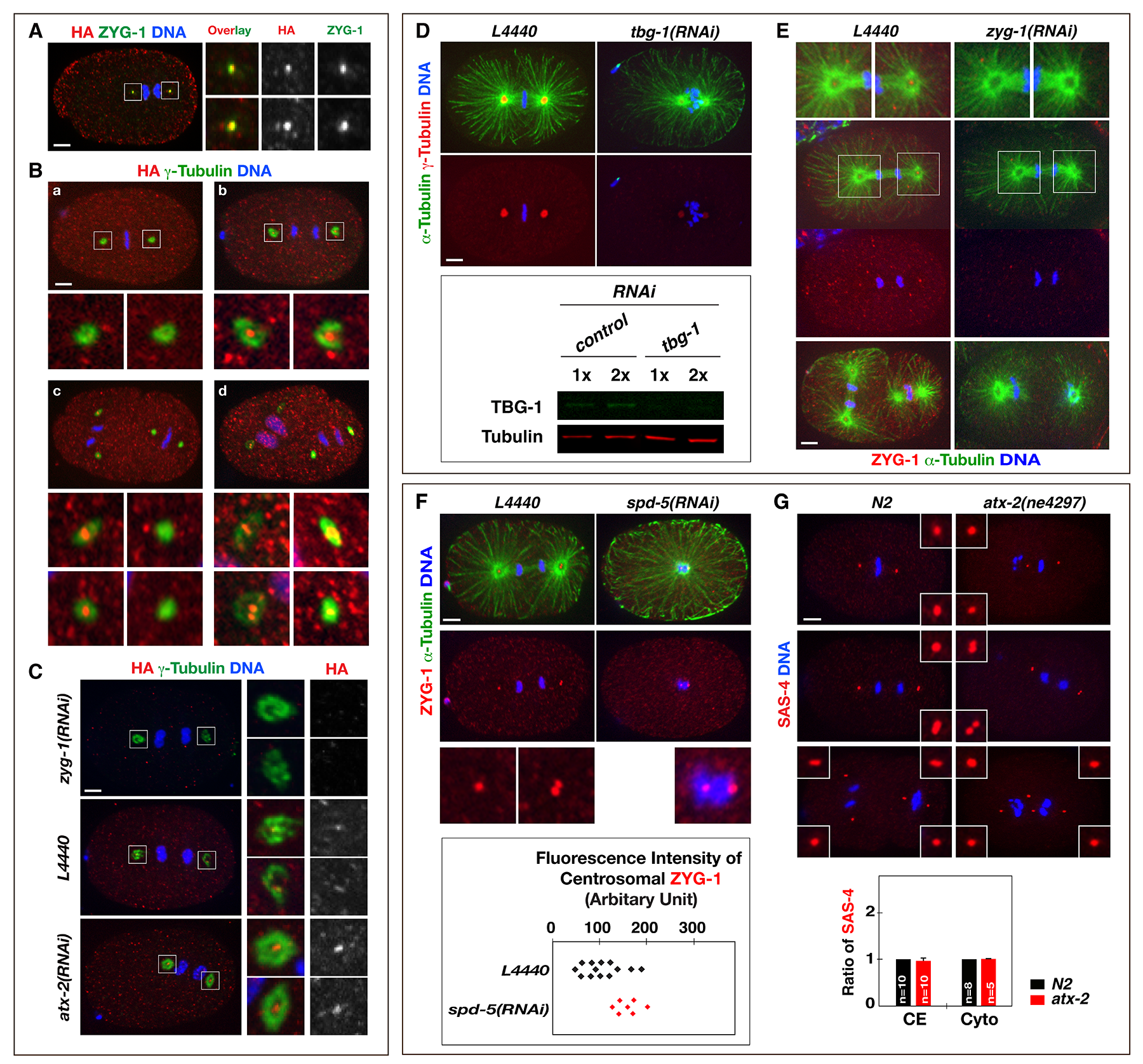

Supplement: S4 Fig — (A-C) The strain expressing an N-terminal HA tagged ZYG-1 at endogenous levels from the native genomic locus is generated using the CRISPR/Cas-9 method (S2 Table for Methods): γ-Tubulin as centrosome marker. HA-ZYG-1 localizes to centrioles, coinciding with anti-ZYG-1 labeling (A) in a cell cycle dependent manner (B): undetectable at first (a) and second (c) metaphase, and highest at late mitosis (b, c, d). (C) zyg-1(RNAi) almost abolishes HA-ZYG-1 signals. Compared to controls, atx-2(RNAi) embryos exhibit increased levels (~2-fold, n = 10) of centrosome-associated HA-ZYG-1 signal at late mitosis. (D) The specificity of γ-Tubulin antibody: Immunostaining and immunoblot reveal that anti-γ-Tubulin specifically detects endogenous γ-Tubulin, as tbg-1(RNAi) leads to a significant reduction in γ-Tubulin signals. (E) The specificity of ZYG-1 antibody: ZYG-1 is enriched at centrioles. zyg-1(RNAi) results in a drastic reduction in both centriolar and cytoplasmic ZYG-1 signals. zyg-1(RNAi) leads to monopolar mitotic spindles at the second mitosis (bottom right). (F) SPD-5 is not required for ZYG-1 localization at centrosomes. ZYG-1 localizes to centrosomes in spd-5(RNAi) embryos that exhibit cell cycle arrest at the first mitosis (Hamill et al., 2002). Quantification of ZYG-1 levels at centrosomes reveals no significant change in ZYG-1 levels by loss of spd-5: Mitotic arrest in spd-5(RNAi) embryos is likely to contribute to more frequent detection of higher fluorescence intensity as ZYG-1 levels at centrosomes peak at late mitosis. (G) SAS-4 levels are not affected by loss of atx-2: SAS-4 stained embryos display SAS-4 localization at early cell cycle stages. Note cell cycle defects (e.g., DNA segregation, spindle positioning, cytokinesis) in atx-2 mutants. Quantification of SAS-4 signals shows that atx-2 mutant embryos exhibit similar levels of SAS-4 at centrosomes (CE) and cytoplasm (Cyto) to those of wild-type embryos at metaphase. Insets are magnified 3-fold. Bar, 5μm. (TI [file pgen.1006370.s004.tif]

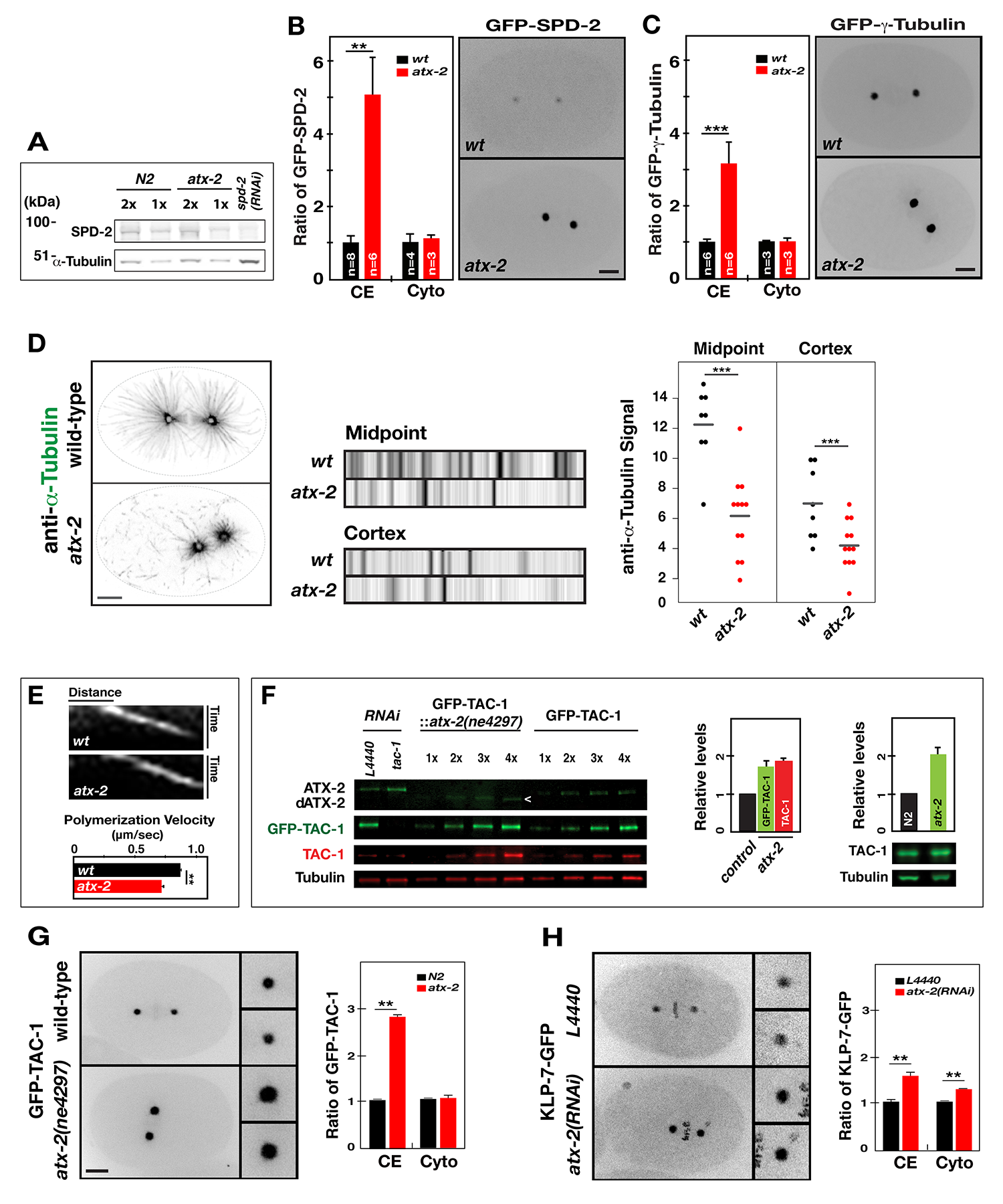

Supplement: S5 Fig — Centrosomal SPD-2 levels (A) are significantly increased without affecting overall levels (B) in atx-2 embryos. (C) atx-2(RNAi) embryos display a nearly 3-fold increase in centrosomal GFP-γ-Tubulin but no change in cytoplasmic levels. (D) Line scans of the kymographs on embryos immunostained for MTs show that atx-2 mutant embryos exhibit fewer MT-signals at the midpoint (9.6 μm away from centrosomes) and near the cortex (1.5 μm inside cortex) compared to wild-type controls, which is consistent with the analyses performed using EBP-2-GFP in Fig 6. Each dot on the graph represents an embryo. Horizontal bars indicate average values, ***p<0.001. (E) Polymerization velocity: Kymographs created along the growing MT. Lines (4.5 μm) were drawn along the individual EBP-2-GFP track extending outward to the cortex. Astral MT growth rates are significantly lower in mutant embryos (0.70 μm/sec ± 0.007) than in controls (0.88 μm/sec ± 0.013, **p = 0.0014). (F) atx-2 mutant embryos exhibit increased levels of TAC-1. Quantitative immunoblot analysis using embryonic extracts of wild-type and atx-2(ne4297) mutants expressing GFP-TAC-1 [73] with α-GFP and α-TAC-1 [72] shows that mutant embryos possess increased levels of both GFP-TAC-1 and endogenous TAC-1. Additional analysis using wild-type and atx-2 embryos confirms that atx-2 embryos contain elevated levels of TAC-1 (n = 5). < indicating a truncated form of ATX-2 (dATX-2) in atx-2 mutants. (A, F) Tubulin as loading control. (G) Z-projections of embryos expressing GFP-TAC-1 at first metaphase. The atx-2 mutant embryo displays a 3-fold increase (**p<0.001) in centrosomal GFP-TAC-1 levels. (H) Z-projections of embryos expressing KLP-7-GFP at first metaphase. In atx-2(RNAi) embryos, KLP-7-GFP signal at centrosomes (122.75 ± 27.72, n = 8) is increased by ~2-fold compared to controls (71.32 ± 13.37, n = 10, **p< 0.001). Kinetochore-associated KLP-7 reveals misaligned chromosomes (arrows) in atx-2(RNAi) embryos. (G, H) Centrosome (CE) an [file pgen.1006370.s005.tif]

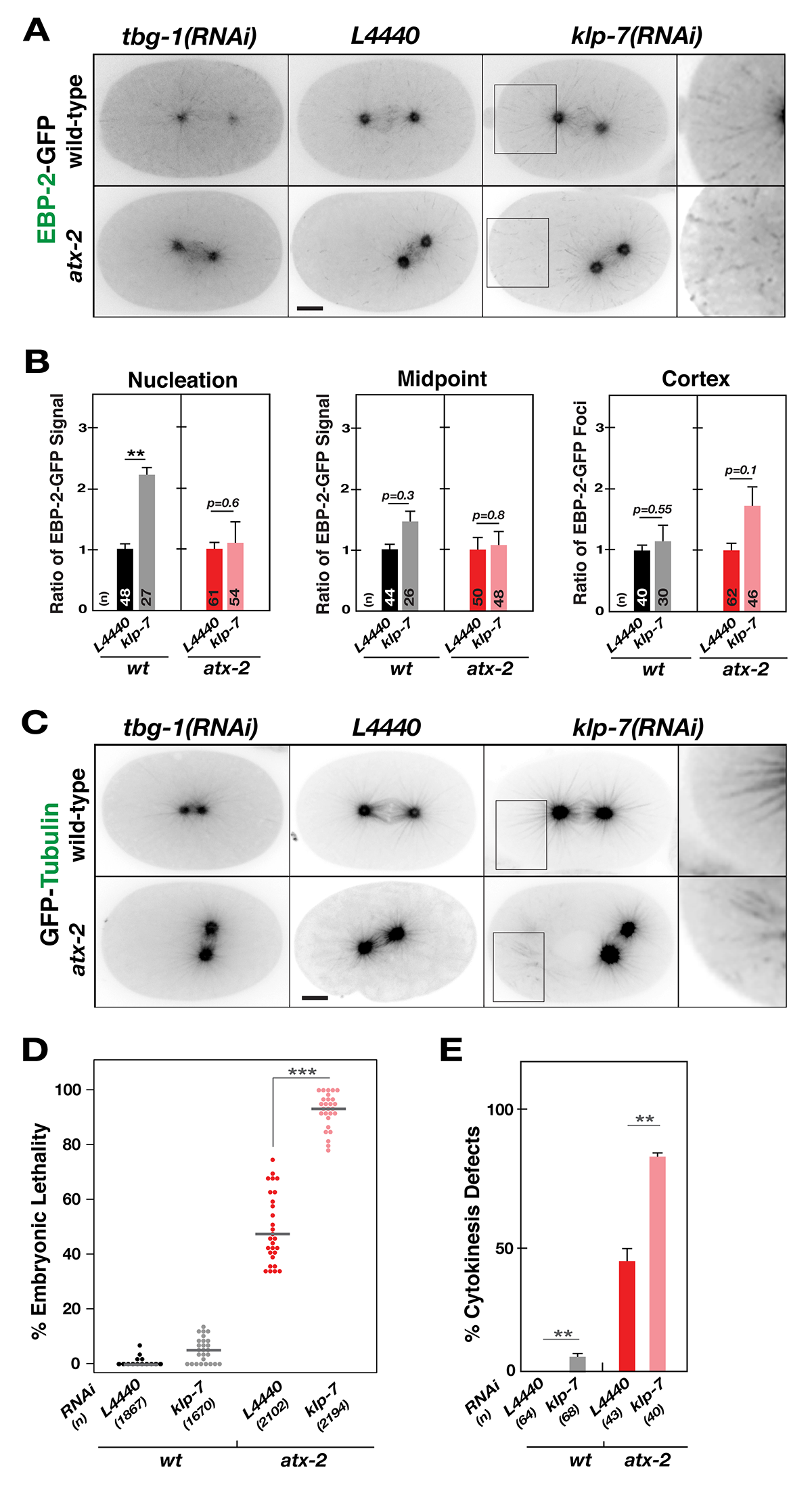

Supplement: S6 Fig — (A) 5-sec time projections of 500 msec interval live imaging of embryos expressing EBP-2-GFP at first metaphase illustrate the effects on MT behavior in RNAi-treated atx-2 mutant, compared to wild-type embryos. tbg-1(RNAi) slightly reduces EBP-2 signals at atx-2 mutant centrosomes whereas tbg-1(RNAi) drastically decreases EBP-2 signals at wild-type centrosomes (n>30), illustrating the effects on MT nucleation by tbg-1(RNAi). In contrast, klp-7(RNAi) increases centrosomal EBP-2 signals in both wild-type and mutant embryos. KLP-7 knockdown also affects cytoplasmic MT growth patterns. Magnified regions illustrate that while MTs emanate from the centrosome and reach the cortex in the wild-type embryo, EBP-2 tracks in mutant embryos appear randomly oriented. (B) Kymograph analysis in klp-7(RNAi) vs control treated wild-type and atx-2 mutants: Levels of EBP-2-GFP signals relative to control RNAi are presented for wild-type and atx-2 mutants, respectively. While klp-7(RNAi) increases MT nucleation by over 2-fold in wild-type (**p = 0.004), relative increase in MT nucleation by klp-7(RNAi) is much lower (1.2-fold) in control atx-2 mutants (p = 0.6). This difference in MT nucleation by klp-7(RNAi) suggests a limited supply of free tubulins available for MT nucleation by mutant centrosomes. The same trends are observed for the midpoint measurement. Interestingly, relative increase in the number of EBP-2 foci near the cortex is higher (p = 0.1) in atx-2 mutants, unlikely due to lengthened MTs growing out from centrosomes. (C) Embryos expressing GFP-Tubulin reveal that in klp-7(RNAi) in the wild-type embryo, MT nucleation is increased and MTs appear to grow longer, originating from centrosomes. However, while more MTs (based on the GFP-Tubulin signal) are found in the cytoplasm distant from the centrosome in klp-7(RNAi); atx-2 embryos compared to L4440; atx-2 embryos, these MTs seem to be disorganized and appear disconnected from the centrosome, which might explain the increase [file pgen.1006370.s006.tif]
