# Supplementary material for: ATX-2, the C. elegans Ortholog of Human Ataxin-2, Regulates Centrosome Size and Microtubule Dynamics
Source: PLoS Genet. 2016 Sep 30;12(9):e1006370. doi: 10.1371/journal.pgen.1006370 (PMC5045193; doi:10.1371/journal.pgen.1006370)
Supplement: S1 Table — (DOCX) [file pgen.1006370.s014.docx]

**S1 Table.** **List of** ***C. elegans s*trains in this study**

| Name | Genotype | Origin |
| --- | --- | --- |
| N2 |  | CGC |
| WM210 | *atx-2(ne4297) III* | *Mello/Shirayama Lab* |
| OC14 | *zyg-1(it25) II* | Kemphues et al. (1988). *Genetics* **120**: 977-986 |
| OC133 | *szy-20(bs52) II* | Kemp et al. (2007). *Genetics* **176**(1):95-113 |
| OC196 | *szy-20(tm1997) II* | Song et al., (2008) *Dev. Cell* **15**(6): 901-912 |
| OC341 | *unc-119(ed3) III; bsIs8[pMS5.1:unc-119(+) pie-1-gfp-zyg-1C-terminus]* | Peters et al. (2010). *J. Cell Sci.* **123**(5):795-805 |
| TH66 | *unc-119(ed3) III; [pie-1::ebp-2::GFP]* | Srayko et al. (2005). *Dev. Cell* **9**(2): 223-236 |
| SA250 | *tjIs54[pie-1p::GFP::tbb-2 + pie-1p::2xmCherry::tbg-1 + unc-119(+)]. tjIs57[pie-1p::mCherry::his-48 + unc-119(+)]* | Toya et al. (2010). *Methods Cell Biol.* **97**:359-372 |
| JH2338 | *unc-119(ed3) III; axls1489[pCG61]* | Gallo et al. (2008) *Developmental Biology* **323(1)**:76-87 |
| MAS15 | *unc-119(ed3); [pie-1::unc-119(+) + pie-1::gfp::klp-7]* | Han et al., (2015) *PLoSOne* **10**(7):e0132593 |
| JA1334 | *weIs11[unc-119(+) + TAC-1::GFP]* | Le Bot et al. (2003)  *Current Biology*  **13:** 1499-1505 |
| MTU 1 | *unc-119(ed3); [unc-119(+) + szy-20 ::gfp::3xflag]* | This study; Sarov et al., (2012) *Cell* **150**(4):855-866 |
| MTU 2 | *unc-119(ed3); [unc-119(+) + atx-2 ::gfp::3xflag]* | This study; Sarov et al., (2012) *Cell* **150**(4):855-866 |
| MTU 3 | *unc-119(ed3); [unc-119(+) +rpn-12 ::gfp::3xflag]* | This Study; Sarov et al., (2012) *Cell* **150**(4):855-866 |
| MTU 4 | *HA::zyg-1(II)* | This Study (**S2 Table**); Dickinson et al., (2015) *Genetics* **120**: 1035-1075; Paix et al., (2015) *Genetics* **201**: 47-54. |
| AZ244 | *unc-119(ed3); [pie-1::unc-119(+) + pie-1::gfp::tubulin]* | Praitis et al., (2001) *Genetics* 157(3):1217-26 |
| TH27 | *unc-119(ed3); [pie-1p::gfp::tbg-1 + unc-119(+)]* | Hannak et al., (2002) *J. Cell Biol.* **157**:591-602 |
