# Supplementary material for: ATX-2, the C. elegans Ortholog of Human Ataxin-2, Regulates Centrosome Size and Microtubule Dynamics
Source: PLoS Genet. 2016 Sep 30;12(9):e1006370. doi: 10.1371/journal.pgen.1006370 (PMC5045193; doi:10.1371/journal.pgen.1006370)
Supplement: S2 Table — (DOCX) [file pgen.1006370.s015.docx]

**S2 Table.** **CRISPR/Cas9-mediated generation of transgenic strains**

Using the CRISPR/Cas-9 method (Dickinson et al., 2013; Paix et al., 2015), we generated a strain expressing HA tagged ZYG-1 at the endogenous level from the N-terminus of the native genomic locus. Cas9/sgRNA plasmids were generated for both *dpy-10* and *zyg-1* using the plasmid pDD162 (Addgene) following the protocol (Dickinson et al., 2015). Single-stranded oligonucleotide (ssODN) homologous repair templates (HRTs) were designed for both *dpy-10(cn64)* (Arribere et al., 2014; Paix et al 2015) and HA::*zyg-1*(codon optimized, IDT)*.* Injections were carried out as described in Dickinson et al., 2015 using the following injection mix (final volume = 20 μL, stock solutions in brackets):

dpy-10 HRT [500 ng/μL]: 0.55 μL

zyg-1 HRT [2 μg/μL]: 1.1 μL

dpy-10 sgRNA plasimd [400 ng/μL]: 2.5 μL

zyg-1 sgRNA plasimd [400 ng/μL]: 2.5 μL

Sterile ddWater: 13.35 μL

Screening was carried out as described by Paix et al., 2015 - jackpot roller/dumpy broods were identified and screened for HA insertion by single-worm PCR and restriction digestion with HpyCH4IV (NEB).

**sgRNA targets used in this study (PAM domain omitted):**

*zyg-1* (5’ end) TCAACTATGAGATGAGCGGT

*dpy-10*  GCTACCATAGGCACCACGAG (From Paix et al., 2015)

**ssODNs used in this study:**

*zyg-1* (HA sequence underlined) tatttttagcgcaccaagtgttgatcaactatgagATGTACCCGTATGACGTGCCTGATTACGCTatgagcggtgggaagagtggttcaagattgagtgtagg

*dpy-10* (From Arribere et al., 2014)

CACTTGAACTTCAATACGGCAAGATGAGAATGACTGGAAACCGTACCGCATGCGGTGCCTATGGTAGCGGAGCTTCACATGGCTTCAGACCAACAGCCTAT

**Mutagenic primers for Cas9/sgRNA plasmid generation:**

*zyg-1* sgRNA mutagenic primer:

TCAACTATGAGATGAGCGGT GTTTTAGAGCTAGAAATAGCAAGT

dpy-10 sgRNA mutagenic primer:

GCTACCATAGGCACCACGAG GTTTTAGAGCTAGAAATAGCAAGT

**HA-*zyg-1* primers for single-worm PCR:**

Forward: GAAACGAGAGACGCAGAG

Reverse: CGCACAACTCCATGACTATG

**Worms generated from this study:** MTU 4 (HA::*zyg-1*)
